# Targeted haplotyping in pharmacogenomics using Oxford Nanopore Technologies’ adaptive sampling

**DOI:** 10.3389/fphar.2023.1286764

**Published:** 2023-11-13

**Authors:** Koen Deserranno, Laurentijn Tilleman, Kaat Rubben, Dieter Deforce, Filip Van Nieuwerburgh

**Affiliations:** Laboratory of Pharmaceutical Biotechnology, Faculty of Pharmaceutical Sciences, Ghent University, Ghent, Belgium

**Keywords:** pharmacogenomics, oxford nanopore technologies sequencing, targeted sequencing, haplotyping, star-allele calling

## Abstract

Pharmacogenomics (PGx) studies the impact of interindividual genomic variation on drug response, allowing the opportunity to tailor the dosing regimen for each patient. Current targeted PGx testing platforms are mainly based on microarray, polymerase chain reaction, or short-read sequencing. Despite demonstrating great value for the identification of single nucleotide variants (SNVs) and insertion/deletions (INDELs), these assays do not permit identification of large structural variants, nor do they allow unambiguous haplotype phasing for star-allele assignment. Here, we used Oxford Nanopore Technologies’ adaptive sampling to enrich a panel of 1,036 genes with well-documented PGx relevance extracted from the Pharmacogenomics Knowledge Base (PharmGKB). By evaluating concordance with existing truth sets, we demonstrate accurate variant and star-allele calling for five Genome in a Bottle reference samples. We show that up to three samples can be multiplexed on one PromethION flow cell without a significant drop in variant calling performance, resulting in 99.35% and 99.84% recall and precision for the targeted variants, respectively. This work advances the use of nanopore sequencing in clinical PGx settings.

## 1 Introduction

Personalizing drug therapy by implementing pharmacogenomics (PGx) holds the promise of achieving better therapeutic outcomes and fewer adverse effects. PGx studies the impact of interindividual genomic variation on drug response, providing the opportunity to tailor the dosing regimen for each individual patient. Over 95% of the population harbors a genetic variation in at least one actionable PGx gene, commonly denoted as pharmacogene, illustrating the large potential of PGx (Ji et al., 2016; Van Der Wouden et al., 2020; Swen et al., 2023). Recently, the results of the European pre-emptive Pharmacogenomic Testing for Preventing Adverse Drug Reactions (PREPARE) study, the largest study on the clinical utility of pre-emptive genotyping up to now, provided convincing evidence for the clinical utility of panel-based pharmacogenetic testing (Van Der Wouden et al., 2020; Swen et al., 2023). Furthermore, PGx information is available on the drug labels of over 360 listed drugs^1^. To facilitate the translation of the observed genetic variation towards clinical use, the Pharmacogene Variation Consortium (PharmVar) uses star (*) designations (Gaedigk et al., 2018; Gaedigk et al., 2021). Based on this nomenclature, gene-drug clinical guidelines have already been issued by the Clinical Pharmacogenetics Implementation Consortium (CPIC) and the Dutch Pharmacogenetics Working Group (DWPG) on how genetic test results can be implemented to improve drug therapy. As germline genetic testing, especially at early adult age, delivers information of livelong value, targeted pre-emptive testing of relevant PGx genes is proposed to be implemented in routine healthcare.

In recent years, there’s been a shift in PGx testing platforms from polymerase chain reaction (PCR)- and array-based methods to massively parallel sequencing (MPS) techniques (Tafazoli et al., 2021a). By adopting MPS for PGx, there’s an effort to address the limitations of traditional methods. This approach not only investigates beyond the commonly known alleles but also offers insights into copy number variations (CNVs). As described previously, even dedicated PGx microarrays failed to detect 25% of the clinically annotated variants in the Pharmacogenomics Knowledge Base (PharmGKB) database (Tilleman et al., 2019). Similarly, the PREPARE study only targeted 50 germline variants in 12 genes using the PCR- based LGC SNPline workflow. For reasons of cost-effectiveness, targeted MPS sequencing of relevant PGx panels or whole exome sequencing (WES) is mostly performed. However, WES misses variants in non-coding and regulatory regions (Tilleman et al., 2020). Alternatively, whole genome sequencing (WGS) has been proposed. As the costs for short-read MPS are continuously decreasing, it is foreseeable that WGS will replace WES in most settings (Tafazoli et al., 2021b). Yet, the magnitude of data originating from WGS results in the need for more computational requirements. Additionally, ethical questions arise about how to handle sequencing data that is not directly relevant to PGx (Johnson et al., 2020).

Furthermore, short-read MPS is still limited in the information it provides for clinical PGx utility. As in short-read sequencing the read length is limited to 600 bp, conventional MPS fails to provide unambiguous haplotype phasing information, and the occurrence of larger structural variants (SVs) can go undetected. Due to the larger genomic regions involved, SVs affect more base pairs per individual than single nucleotide variants (SNVs). In addition, highly similar genes, such as the *CYP2D6* gene and the highly similar neighboring *CYP2D7* and *CYP2D8* pseudogenes, are difficult to characterize using short-read sequencing due to mapping issues (Nofziger and Paulmichl, 2018; Tafazoli et al., 2021b).

It is clear that being able to identify all genomic variants is required to make comprehensive statements of the resulting phenotype. Long-read sequencing (LRS) has been used before for PGx purposes to deliver information on SNVs, SVs, and haplotype phasing in complex loci. Their potential for pre-emptive clinical testing has been confirmed (Fukunaga et al., 2021; van der Lee et al., 2021; Scott et al., 2022; van der Lee et al., 2022). In recent years, the accuracy of the two leading LRS systems has been substantially increased, with Oxford Nanopore Technologies’ Q20+ chemistry and PacBio’s HiFi long-read sequencing. However, most targeted applications using these sequencing platforms rely on long-range PCR to amplify the regions of interest before sequencing (Liau et al., 2019). This PCR-step has been clearly shown to introduce artificial chimeras which could result in downstream haplotyping errors (Ammar et al., 2015; Laver et al., 2016). Recently, the PCR-free nanopore Cas9-targeted sequencing (nCATS) method was successfully used to characterize the full *CYP2D6-CYP2D7* locus to overcome this problem (Gilpatrick et al., 2020; Rubben et al., 2022). However, optimization is still required and the iterative, time-intensive process of guide RNA design and testing should be repeated for each locus of interest of the PGx panel individually.

Therefore, we harnessed ONT’s adaptive sampling enrichment feature to target the relevant PGx genes extracted from PharmGKB (Whirl-Carrillo et al., 2021). Thanks to the real-time sequencing nature of ONT sequencing, pre-selected DNA-molecules can be accepted or rejected by the sequencer based on fast initial alignment against a reference. This selection results in increased sequencing of DNA of interest without any additional library preparation steps, thereby fully occupying the sequencer’s capacity to sequence target strands. The list of target genes can be rapidly modified, without time-consuming optimization, if new actionable PGx genes would be identified. We hypothesize that the resulting long-read PCR-free information will result in improved characterization of a patient’s haplotype and provide a better prediction of phenotypical consequences in a cost-effective manner.

## 2 Materials and methods

### 2.1 Target gene selection and .bed file construction

The list of relevant PGx genes was constructed based on the clinical_annotations.tsv file downloaded from the PharmGKB database (Whirl-Carrillo et al., 2021). All clinical annotated variants were extracted from the clinical_annotations.tsv file. First, target regions were defined. If a variant was present within the gene body (exon or intron), the entire gene was selected as a target. When a variant was present outside the gene body, the variant was considered of interest if located within 100 kb from the gene’s start or end position. In the latter case, the target region was defined as the span ranging from the start/end of the gene until that variant. This resulted in a panel of 3,347 variants, including 3,262 SNVs and 85 INDELs, linked to 1,036 genes. Secondly, 20 kb was added to each target region’s determined up- and downstream coordinates. This step was needed to account for large DNA-molecules, of which the first nucleotides being sequenced would not correspond to the target region but do contain the target region further in their sequence. Coordinates of overlapping target regions were collapsed into one region. The final .bed file targeted 5.68% of the human genome (Supplementary Table S1). A target region corresponding to 1%–10% of the human genome is recommended by ONT (ONT, 2020).

### 2.2 DNA sample collection and QC

The NA12878 (HG001), HG01190, NA19785, NA24385 (HG002), and NA24631 (HG005) reference DNA standards were obtained from the Coriell Institute for Medical Research (Camden, NJ, United States). The concentration was verified using a PicoGreen assay (ThermoFisher, Waltham, MA, United States) according to the manufacturer’s instructions. The length of the DNA molecules was evaluated on Femto Pulse using the Agilent Genomic DNA 165 kb kit (Agilent Technologies, Santa Clara, CA, United States) according to the manual.

### 2.3 Library preparation

Library preparation for the HG001 reference on the PromethION R9.4.1 flow cell was completed as described in the Ligation sequencing gDNA manual of ONT using the SQK-LSK110 library preparation kit. 1.5 µg of unsheared input DNA was used for DNA repair and end-prep. After adapter ligation and clean-up, 70.5 fmol of the final library was diluted with elution buffer (ONT, Oxford, United Kingdom) and divided into three tubes. The flow cell was initially loaded with 23.5 fmol. After 22 h and then 40 h of sequencing, we washed the flow cell according to ONT’s Flow Cell Wash Kit protocol and loaded the second and third portions of the library.

Library preparation for HG002 and HG005 on the PromethION R10.4.1 flow cell was completed based on the Native Barcoding Kit 24 V14 protocol using the SQK-NBD114.24 kit (ONT, Oxford, United Kingdom). For each reference standard, 4 µg of unsheared DNA was used as input, and the reagent volumes were doubled to yield a larger final library compared to using the standard volumes. After DNA end-repair and clean-up, the concentration of each individual sample was determined using a PicoGreen assay (ThermoFisher, Waltham, MA, United States). Based on the limiting one, an equimolar amount of each sample was used in the barcoding reaction to obtain an equal number of reads for each sample. After barcoding, libraries were pooled and adapter-ligated. After the final clean-up, 79 fmol of the library was obtained. 50 fmol of the library was loaded on the flow cell. After 36 h of sequencing, the library was recovered from the flow cell as stipulated in ONT’s ‘Library recovery from flow cells’ protocol. After washing the flow cell, the recovered library was reloaded on the same flow cell, supplemented with the remaining 29 fmol of the library.

Library preparation for the HG001, HG01190, and NA19785 samples on the PromethION R10.4.1 flow cells was performed similarly as described above. For each reference standard, 3 µg of unsheared DNA input was used, and 180 fmol of final sequencing library was obtained. Initially, the flow cell was loaded with 60 fmol of the library to increase pore occupancy. After 20 h of sequencing, the flow cell was washed and reloaded with 70 fmol of the library. Finally, after 42 h, the flow cell was washed again and reloaded with 50 fmol of the library. As there was still pore capacity remaining after 72 h of sequencing, we performed the Library recovery protocol with an additional 10 µL spike of sequencing buffer and restarted the run.

### 2.4 Data analysis pipeline

The raw .fast5 sequencing data was first rebasecalled using the super-accurate basecaller (SUP) model of Guppy version 6.4.2-gpu for the R9.4.1 and the R10.4.1 HG002/HG005 flow cell and Guppy version and 6.5.7-gpu for the R10.4.1 HG001/HG01190/NA19785 flow cell. Only reads with a quality score above 10 were used for further analysis. Only the reads positively selected by the adaptive sampling (AS) software were used in downstream analysis to avoid using short reads of the rejected strands that often do not map uniquely. Subsequently, the basecalled data were aligned to the GRCh38 reference genome using minimap2 version 2.18-r1015 (Li, 2018). Next, variant calling was performed using Clair3 version v0.1-r10. Variants were phased using WhatsHap version 2.0 with options --include-homozygous --indels --distrust-genotypes. Variant calling was benchmarked using the hap.py software. Recall and precision values were calculated as truth_tp/(truth_tp + truth_fn) and query_tp/(query_tp + query_fp), respectively. According to the best practices for benchmarking small variant calls, truth negative calls are not commonly included due to the high proportion of concordant reference positions in case of WGS (Krusche et al., 2019). However, as in this case we benchmark only the predetermined variants of our panel, the concordant calls are meaningful to report. Indeed, detection of absence of the alternate (ALT) allele is in PGx settings as important as detection of the reference (REF) allele. Therefore, we calculated the accuracy as the (truth_tp + truth_tn)/(total variants present in the truth set). For HG001, the hybrid Genome in a Bottle Consortium (GIAB) - Platinum Genomes benchmark dataset described by Krusche et al*.* was used (Krusche et al., 2019). For HG002 and HG005, the GIAB benchmark version v4.2.1 was used (Wagner et al., 2022a).

For 24 out of the 1,036 genes included in our target .bed file, and defined as Very Important Pharmacogenes (VIPs) by PharmGKB, the *-alleles were determined using Aldy version v4.4 (https://github.com/0xTCG/aldy) (Numanagić et al., 2018; Hari et al., 2023). For HG001, HG01190, and NA19785, the results were compared with the star alleles in the Genetic Testing Reference Material Coordination (GeT-RM) studies. For HG002 and HG005, no *-allele truth set was available, and we provide the results for further reference.

## 3 Results

### 3.1 Sequencing summary statistics and evaluation of variant calling performance

In the R9.4.1 sequencing run, we performed SQK-LSK110 library preparation on the HG001 reference DNA standard and subsequently sequenced the library on a PromethION R9.4.1 flow cell to establish the baseline performance of AS on the PGx panel. This run resulted in an average depth on-target of 47 X and coverage at 20 X of more than 99.9% of the targeted region (Table 1). Of the 3,347 targeted PGx variants selected as described in Methods, only 3,262 are present in the truth set of HG001. Of these, 99.69% were called correctly in this dataset (Table 2).

**TABLE 1:** Summary statistics for the R9.4.1 and R10.4.1 multiplex runs.

	R9.4.1	R10.4.1		R10.4.1		
	HG001	HG002	HG005	HG001	HG01190	NA19785
Gb output	49.9	55.9		73.3		
Average depth on-target	47X	40X	23X	20X	32X	24X
% at 30X	98.8	91.2	11.1	5.1	64.6	17.8
% at 20X	99.9	99.4	71.2	57.7	97.6	81.1
% at 15X	99.96	99.8	93.9	89.1	99.6	97.0

**TABLE 2:** Summary of the variant calling metrics for the targeted variants in the HG001 reference sample relative to the Krusche et *al.* reference using the R9.4.1 flow cell.

	PGx variants in HG001	ALT variants in truth set	ALT variants in R9.4.1 data	Recall (%)	Precision (%)	Accuracy (%)
Variants	3,262	1,229	1,224	99.59	99.59	99.69
SNVs	3,186	1,218	1,214	99.67	99.75	99.79
INDELs	76	11	10	90.91	83.33	96.05
Genes	1,023					

Next, two samples, HG002 and HG005, were pooled to increase cost-effectiveness, using the newly released Q20+ Kit14 chemistry and the R10.4.1 flow cells in early access. An average coverage of 40 X and 23 X was retrieved for HG002 and HG005 on a single flow cell (Table 1). Despite the average coverage being only half of the coverage in the first run, recall of over 99% was obtained for both samples (Table 3).

**TABLE 3:** Summary of the variant calling metrics for the targeted variants in the barcoded HG002 and HG005 reference samples relative to their GIAB references using a single R10.4.1 flow cell.

	PGx variants present	ALT variants in truth set	ALT variants in R10.4.1 data	Recall (%)	Precision (%)	Accuracy (%)
HG002						
Variants	3,229	1,048	1,042	99.43	99.81	99.75
SNVs	3,155	1,039	1,035	99.62	100.00	99.87
INDELs	74	9	7	77.78	77.78	94.59
Genes	1,008					
HG005						
Variants	2,813	1,039	1,030	99.13	100.00	99.68
SNVs	2,762	1,032	1,025	99.32	100.00	99.75
INDELs	51	7	5	71.43	100.00	96.08
Genes	991					

Additionally, two other GIAB samples, HG01190 and NA19785, were multiplexed on another PromethION R10.4.1 flow cell, along with HG001. We chose the latter samples as extensive *-allele nomenclature is available for these samples. While we anticipated that recall and precision scores might drop, we hypothesized that the coverage obtained would still be sufficient to detect larger structural variants. Table 4 summarizes the recall and precision percentages of the SNVs and INDELs compared to their references.

**TABLE 4:** Summary of the variant calling metrics for the targeted variants in the HG001 reference sample relative to the Krusche *et al.* reference using an R10.4.1 flow cell loaded with a pooled library of the barcoded HG001, HG01190, and NA19785 reference samples. As the truth set of SNVs and INDELs for the HG01190 and NA19785 reference samples is not available, direct comparison is not possible.

	PGx variants present	ALT variants in truth set	ALT variants in R10.4.1 data	Recall (%)	Precision (%)	Accuracy (%)
HG001						
Variants	3,262	1,229	1,224	99.35	99.84	99.70
SNVs	3,186	1,218	1,214	99.43	99.92	99.75
INDELs	76	11	10	90.91	90.91	97.65
Genes	1,023					

### 3.2 Structural variant calling and haplotype phasing

WhatsHap was applied for read-based phasing of the identified SNVs, indels and complex variants, as it specifically can make use of the long-read information. The maximum read length that can be achieved using ONT only depends on the length of the input fragments. This results in the ability of reads spanning many variants, including large and complex structural variants. The number of variants phased per gene for each sample is documented in Supplementary Table S3.

As stated before, variation in pharmacogenes is commonly denoted using the *-allele classification. Therefore, we applied Aldy v4.4 to automatically assign *-alleles to the gathered AS sequencing data. Since the performed library preparation does not depend on PCR-amplification, no artificial hybrids are expected. The results are summarized in Table 5.

**TABLE 5:** Comparison of the reference GeT-RM PGx star-allele calls to the calls made by Aldy for the VIP genes. The light blue, dark blue, and orange bars indicate that the call is identical to the reference, augments the reference, or is incorrect, respectively. White bars indicate that there is no reference call available.

	HG001			HG01190		NA19785	
Gene	GET-RM	ALDY R9.4.1	ALDY R10.4.1	GET-RM	ALDY R10.4.1	GET-RM	ALDY R10.4.1
*CFTR*	– (*WT/*WT)^∆^	*WT/*WT	*WT/*WT	–	*WT/*WT	–	*WT/*WT
*COMT*	–	*Met/*ValA	*Met/*ValA	–	*Met/*ValA	–	*Met/*ValB
*CYP1A2*	*1F/*1F	*1M/*1M	*1M/*1M	*1A/*1A	*1B/*1B	*1L/*1L	*1L/*1L
*CYP2A13*	*1A/*1A^≠^	*1/*1	*1/*1	*1A/*1A^≠^	*1/*1	–	*1/*1
*CYP2A6*	*1/*1	*1+*1/*12	*1+*1/*12	*1/*1	*1/*1	*1/*1^≠^	*1/*1
*CYP2B6*	*1/*1	*1/*1	*1/*1	*1(*5)/*1(*27)	*1/*5	*1/*1	*1/*5
*CYP2C19*	*1/*2	*1/*2	*1/*2	*1/*2	*1/*2	*1/*1	*1/*1
*CYP2C8*	*1/*3	*3/*5	*1/*3	*1/*3	*1/*3	*1/*1	*1/*1
*CYP2C9*	*1/*2	*1/*2	*1/*2	*2/*61	*1/*61	*1/*1	*1/*1
*CYP2D6*	*3/*4+*68	*3 + *82/*4 +*132	*4N.ALDY/*10+*82	*4/*5	*4/*4	*1/*2+*13	*2/*13
*CYP2E1*	no consensus (*5)/*7	*1/*5A_7A_1B	*1/*5A_7A_1B	*1/*7	*1/*7	*7/*7^≠^	*4/*5
*CYP2J2*	*1/*1^≠^	*1/*1	*1/*1	*1/*7≠	*1/*7	–	*1/*1
*CYP3A4*	*1/*1	*1/*1	*1/*1	*1/*1B	*36/*36	*1/*1	*1/*36
*CYP3A5*	*3/*3	*3/*3	*3/*3	*1/*1	*1/*1	*1/*3	*1/*3
*CYP4F2*	*1/*1	*1/*1	*1/*1	*1/*3	*1/*3	*3/*3^≠^	*3/*3
*DPYD*	*1/(*4)	*4/*5	*4/*5	*1/*9	*1/*9	*1/*1	*1/*1
*G6PD*	NEG^≠^	*B/*B	*B/*B	NEG≠	*B/*B	NEG^≠^	*B/*B
*GSTP1*	*A/*C; *B/*D	*A/*C	*A/*C	*A/*B	*A/*B	*A/*B^≠^	*A/*B
*NAT2*	*4/*5	*4/*5	*4/*5	*4/*4	*4/*4	no consensus	*7/*7 (curated: *7/*12)
*NUDT15*	– (*1/*1)‡	*1/*1	*1/*1	– (*1/*1)‡	*1/*1	– (*1/*1) ‡	*1/*1
*SLCO1B1*	*1/*15	*1/*15	*1/*15	*1/*1	*1/*1	*1/*1	*1/*37
*TPMT*	*1/*1	*1/*1	*1/*1	*1/*1	*1/*1	*1/*1	*1/*1
*UGT1A1*	*1/*28	*60_80/*112	*1/*28_60_80_93	no consensus (*37)/*60	*1/*60_80 (curated: *1/*37_60_80)	no consensus	*1/*28_60_80_93
*VKORC1*	H1/(H9)	*H1/*H8	*H1/*H9	*H7/*H7	*H7/*H7	*H1/*H1	*H1/*H1

∆ Diplotype not determined in Get-RM, based on the results of Pranesh et al. (2019), *WT/*WT, was assigned.≠ Diplotype based on Get-RM, non-consensus data (data from one assay only). For G6PD, NEG, denotes the absence of the A+/A-allele in the Tech Open Array. *B/*B outputted by Aldy denotes wild type.‡ Diplotype not determined in Get-RM, based on the results of Yaqing *et al.* (Liu et al., 2023), the diplotype was assigned.

## 4 Discussion

We applied the AS feature on PromethION to *in silico* enrich for a panel of 1,036 PGx genes to assess its potential for personalizing drug treatment regimens. PGx testing panels are already incorporated in clinical standard-of-care settings in some of the major hospitals worldwide, e.g., the Erasmus MC hospital in the Netherlands offers six Taqman probe-based PGx testing panels. These are available upon request by general practitioners or pharmacists. However, the number of variants assayed per gene is limited^2^. In other settings, more comprehensive tests are performed. The American College of Medical Genetics and Genomics (ACMG) recently issued an update of their technical standard recommendations for clinical PGx testing and reporting (Tayeh et al., 2022). These recommendations include aspects for both targeted PGx testing, WES/WGS, and CNV testing. 34.49% (269/780) of the PGx tests currently listed in the United States National Institute of Health Genetic Testing Registry (GTR) are classified as assays involving sequence analysis of the entire coding region (Rubinstein et al., 2012). Still, variants residing in intronic or regulatory regions remain excluded, despite their potential functional consequences in, e.g., *CYP2C19* or *CYP3A4* (Morales-Rosado et al., 2021; Zhou et al., 2022). As our AS strategy goes beyond targeted PGx testing for specific variants and inherently encompasses CNV information, we improve the current state-of-the-art genotyping assays.

The panel we utilized encompasses the genes with demonstrated PGx relevance, as evidenced by their inclusion in PharmGKB. We also incorporated genes with variants of lesser evidence, anticipating that these might gain higher evidential status as more data emerges. For each gene, we examine the entire gene locus, which includes intronic regions and areas up to 20 kb both upstream and downstream of the starting coordinates. Thanks to the flexibility in the way the target file is constructed, our *in silico* AS enrichment strategy easily permits to include new genes as new medically relevant information becomes available.

### 4.1 Recall and precision assessment for HG001, HG002, and HG005

For the GIAB HG001 reference sample on the R9.4.1 flow cell, overall recall and precision for the variants in the PharmGKB database were 99.59% and 99.59%, respectively. These numbers are comparable to recent whole genome sequencing results reported by Byrska-Bishop et al. (Byrska-Bishop et al., 2022), i.e., recall of 99.53% and precision of 99.57%, generated using Illumina NovaSeq sequencing targeting 30X whole genome coverage and the GATK HaplotypeCaller.

In the subsequent run, we multiplexed the HG002 and HG005 samples on a single R10.4.1 flow cell using ONT’s latest chemistry. We hypothesized that the increased read accuracy of the newest chemistry would permit to accurately genotype two samples simultaneously, even at the anticipated lower coverage than for the former non-multiplexed sample. Indeed, recently it was shown that assuming a Poisson coverage distribution, 6 X and 8 X coverage suffices to recall 98% and 90% of the homozygous and heterozygous variants, respectively (Mahmoud et al., 2023). As illustrated in Table 3, we obtain similar sensitivity and even higher precision scores than for HG001. Recently, Wagner et al. defined an additional benchmark for 273 autosomal challenging medically relevant genes (CMRG) in HG002, from which less than 90% of the bases were included in the latest GIAB v4.2.1 benchmark, as they are challenging to sequence or contain challenging variants (Wagner et al., 2022b). 32 of the 1,036 genes targeted in our set-up are present in the CMRG benchmark. Benchmarking variant calls for these 32 genes to the CMRG-reference resulted in the perfect recall and precision scores for all INDELs and SNVs present in PharmGKB.

In the final experiment, we checked the ability to multiplex three samples, HG001, HG01190, and NA19785 on a single R10.4.1 flow cell. We chose these three GIAB samples to compare the recall and precision for HG001 with the R9 flow cell data without multiplexing. Secondly, extensive *-allele reference calls are available for these samples. Recall and precision values are shown in Table 4. Overall recall for the variants included in the PharmGKB panel dropped only by 0.24% while average depth decreased from 47 X to 20 X. Precision increased from 99.59% to 99.84%. We illustrate that the transition from the R9.4.1 to the R10.4.1 flow cell resulted in clear benefits for variant calling in PGx regions. The latter flow cells are now also considered as default by ONT. Therefore, we conclude that multiplexing of three samples on a single R10.4.1 PromethION flow cell while enriching for 5.68% of the human genome is acceptable for PGx.

Multiplexing three samples on a single flow cell allows the estimated cost per sample to drop to € 320 (Supplementary Table S2). Interestingly, Twist Bioscience recently launched a long-read probe-based PGx panel, optimized for PacBio sequencing. However, this panel only targets 49 genes and requires new probe design if novel targets have to be included. In addition, the capture reaction and accompanying reagents add to the final cost^3^.

### 4.2 Long-read phasing success rate

**FIGURE 1:**
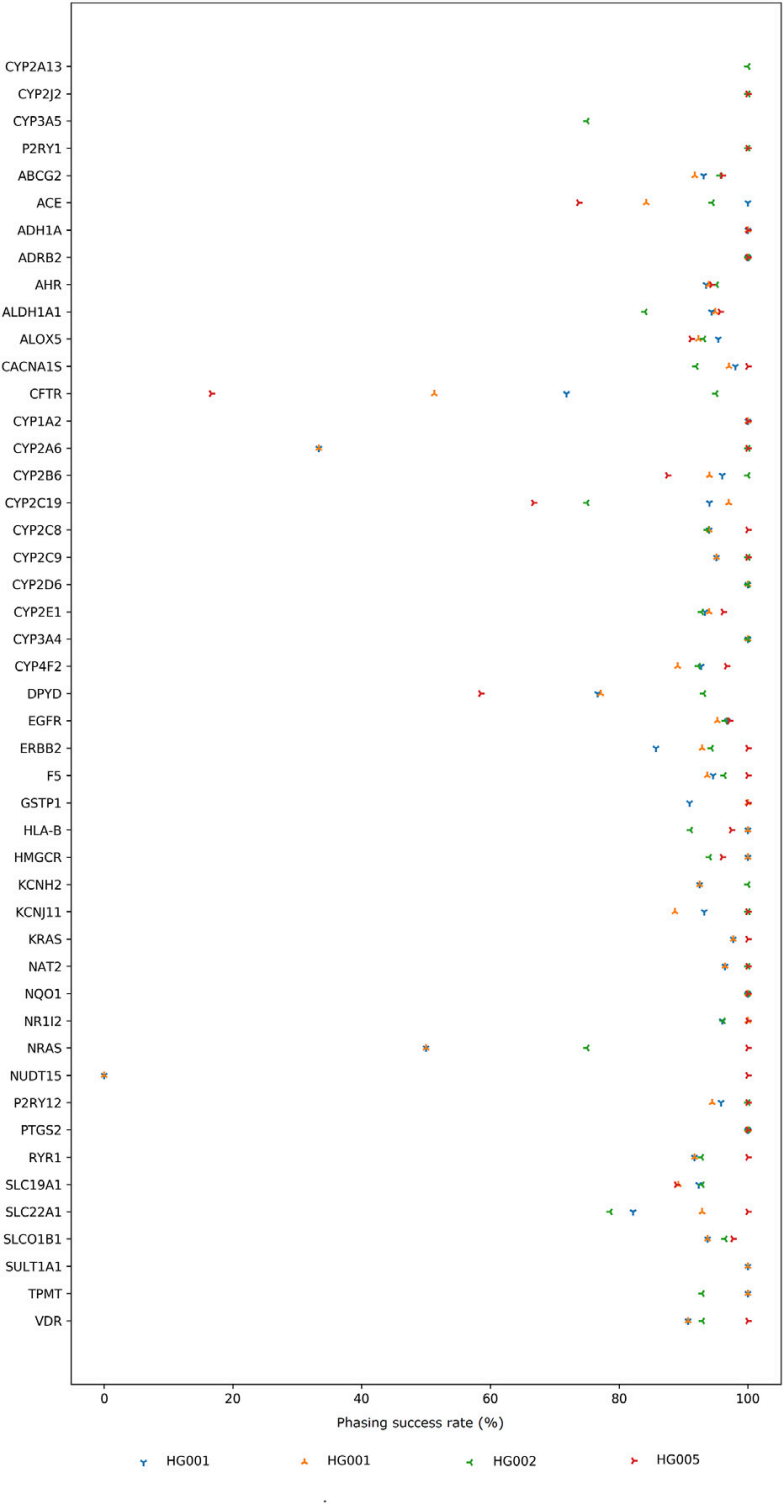
Percentage of variants that were correctly phased compared to their respective truth sets. For figure clarity, the percentages are only shown for 47 Very Important Pharmacogenes (VIPs) for which reference phasing information was available. The number of reference variants considered for each gene in each sample can be retrieved from Supplementary Table S3.

We used our long-read data to phase both alleles, which is crucial for accurate *-allele assignment. While short-read, imputation-based methods are not ideal for informing PGx at an individual patient level, long reads allow for the direct phasing of both alleles without the need for additional population or trio data. Based on the ground truth set of phased variants for HG001, HG002, and HG005, we calculated the phasing success rate for 47 PharmGKB VIPs for which reference phasing information was available (Figure 1). For some genes, e.g., *CFTR*, *CYP2A6*, *CYP2C19*, *NRAS*, *NUDT15*, clear differences in the phasing success rate between the samples are observed. While discrepancies for the HG001 sample on the R9 and R10 flow cell can be attributed to differences in sequencing depth and chemistry, large differences between samples are most likely the result of the number of heterozygous variants available for phasing. For example, the *CFTR* gene in the HG001 reference encompasses 39 phased variants (Supplementary Table S3). In contrast, the HG002 reference encompasses 280 phased variants for the same gene. The more heterozygous variants inherently present in the reference sample, the larger the haplotype blocks that can be constructed based on our sequencing data and the higher the odds of phasing more variants. Additionally, the impact of unphased variants on the calculated phasing success rate is higher if the reference only considers a limited number of variants as demonstrated for *CYP2A6*, i.e. 1 variant out of the 3 considered reference variants was correctly phased in our HG001 data compared to 1 variant out of the 1 considered reference variant correctly phased in our HG002 data (Supplementary Table S3).

**FIGURE 2:**
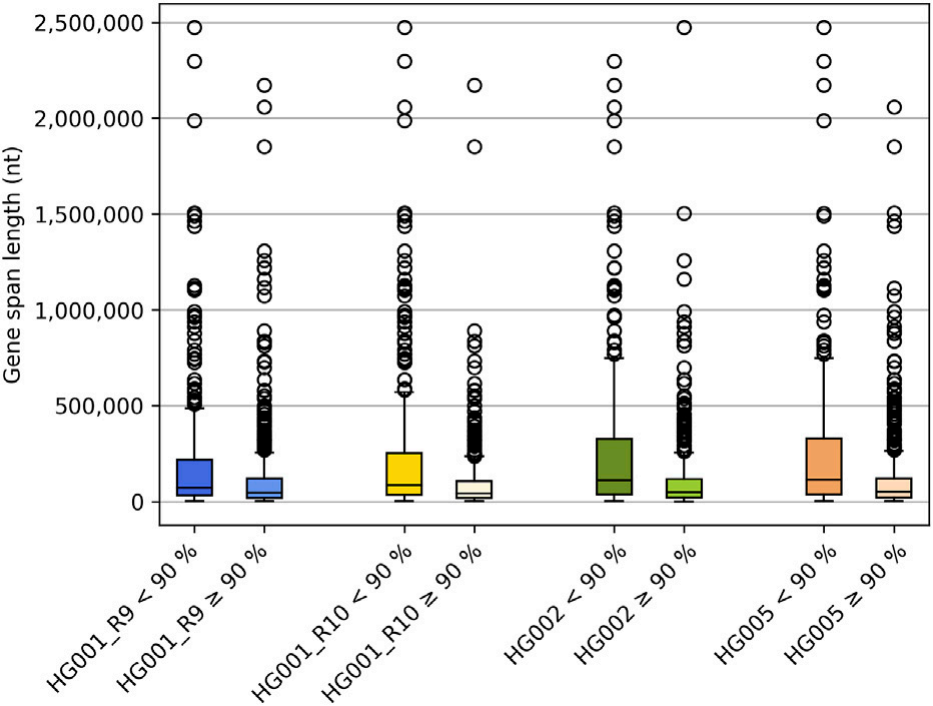
Boxplots illustrating the gene span lengths for genes for which less or more/equal to 90% of the variants could be traced back to their allele of origin, respectively.

As expected, genes encompassing very large genomic regions contain more unphased variants than smaller genes, as even the long reads do not permit to span the entire gene locus. Upon comparing the gene span length between genes for which either less or equal to/over 90% of the variants could be phased correctly (Supplementary Table S3), our results clearly indicate the difference in length between both. Figure 2 depicts the relationship between gene span length and the number of genes that are either less than or over 90% phased across various reference samples. These findings highlight the significance of using high-molecular-weight DNA as the input material. Longer reads increase the likelihood of capturing more variants within that read, thus extending the haplotype phasing block. Supplementary Figure S1,S2 show that the average input DNA-length used varied between reference samples. Dedicated high-molecular weight DNA-extraction protocols might be required as DNA extracted for short-read NGS or microarrays may not be of sufficient quality and length for LRS PGx purposes.

### 4.3 Star allele calling using Aldy 4

Finally, we used Aldy 4 to perform *-allele calling of the PGx genes in our panel (Numanagić et al., 2018; Hari et al., 2023). For optimal utilization of the PGx information in our dataset, we need an analysis tool that incorporates SNV, INDEL, and CNV variants, as well as the phasing information provided by the long reads. While most tools like PharmCAT use .vcf files as input for *-allele calling, and subsequently ignore copy number variants and gene fusions, Aldy 4 accepts whole genome .bam files as input (Sangkuhl et al., 2020). The PyPGx package has a pipeline dedicated to long-read sequencing data using .vcf files as input, but does not support structural variant detection (Lee et al., 2022). Although tools such as Cyrius also use a WGS .bam file, they are designed for short-read data and are specific to the *CYP2D6* locus (Chen et al., 2021). The benchmarking results from Graansma *et al.* and Shugg *et al.* strengthen us in using Aldy as the most informative tool (Graansma et al., 2023; Shugg et al., 2023). We summarized our results for the 24 VIPs that Aldy currently supports in Table 5.

The GeT-RM calls for HG001, HG01190, and NA19785 were used as a reference, supplemented with other available sources if the former did not provide reference information (Pratt et al., 2016; Gaedigk et al., 2019; Gaedigk et al., 2022). While Aldy can call both major and minor alleles, only major *-alleles were considered, as no minor allele calls are available from GeT-RM. Nevertheless, the raw Aldy output files containing minor allele calls are provided in Supplementary Table S4–S9.

For the R9.4.1 data, there were 14 concordant calls, 8 discordant calls, and 2 calls without a reference. However, for 3 of these discordant calls, more specifically for *CYP1A2*, *DPYD*, *GSTP1,* our results improved the reference calls. For *CYP1A2*, the *1M allele was not included in the GeT-RM, and *1M allele shares the rs762551 variant with *1F. However, our results clearly indicate the presence of the additional rs2472304 for both alleles (Supplementary Figure S3). For *DPYD,* *4 was only found by Affymetrix DMET, LifeTech Taqman LDT, and Agena Bioscience iPLEX ADME PGx Pro did not check for it. None of the reference assays assessed *5 (Supplementary Figure S4). For *GSTP1,* both Affymetrix and Agena could not discriminate between *A/*C or *B/*D, nor could Ramudo-Cela L. et al. using an NGS panel (Ramudo-Cela et al., 2020). Aldy miscalled the *CYP2A6* diplotype by assuming the presence of the *12 structural variant. For *CYP2C8*, *UGT1A1* and *VKORC1*, the discordant results can be explained by the presence of an underlying homopolymeric region, known to be problematic for the ONT R9.4.1 pore (Stevanovski et al., 2022). Lastly, the incorrect call for the *CYP2D6* *4+*68 haplotype is anticipated, as this haplotype was also missed in the Aldy publication (Hari et al., 2023).

The calls made based on the R10.4.1 data are correct for *CYP2C8*, *VKORC1,* and *UGT1A1*, despite the reduced coverage. This is not unexpected, as ONT has implemented a dual reader head in the R10 pores, to augment basecalling in homopolymeric regions.

For HG01190 on the R10.4.1 flow cell, our results improve the calls made for the *CYP1A2*, *CYP2B6*, *CYP2C9*, and *CYP3A4* genes while no correct Aldy calls could be made for *CYP2D6* and *UGT1A1*. For *CYP1A2*, the *1B allele was not examined in the GeT-RM studies so the presence of this allele was manually verified (Supplementary Figure S5). For *CYP2B6,* LifeTech Taqman LDT and Agena Bioscience iPLEX ADME PGx Pro found *1/*1 but did not check for *5. Affymetrix found *5 but called the other allele as *27 as it incorrectly assumed the presence of the rs36079186 variant. The *1/*61 call compared to the *2/*62 call of the GeT-RM NGS panel for *CYP2C9* was also reported by *Liu Y. et al* (Liu et al., 2023). For *CYP3A4*, *36 was not assessed in the GeT-RM studies. Aldy is correct in assigning *36/*36, however, this haplotype was withdrawn from PharmVar as of version v5.2.17. After manual curation according to the latest version of PharmVar, this allele should now be noted as *1/*1.

For NA19785, the improved calls for *CYP2B6* and *SLCOB1* are concordant with the results of *Liu Y. et al* (Liu et al., 2023). For *CYP2E1*, the Agena assay did not examine *4 nor *5, while the variants were manually validated to be present in IGV (Supplementary Figure S6). Aldy is correct in *1/*36 for *CYP3A4*, however, as mentioned before, *36 of *CYP3A4* is withdrawn and this haplotype should now be denoted as *1. For *NAT2*, Aldy calls *7/*7, but after manual curation, *7/*12 should have been assigned (Supplementary Figure S7). Due to low coverage, Aldy calls *7 for the *12 allele as it assumes that rs1799931 is present on this allele. As for the other GIAB samples, Aldy fails to correctly diplotype *CYP2D6*.

*CYP2D6* *-allele calling consistently failed for the tested reference samples, which can be attributed to the complex genomic context of the gene. In HG01190, Aldy miscalled one allele as *4 instead of *5 (full gene deletion) probably due to misalignment of the *CYP2D7*-mapping reads to *CYP2D6* for that allele. Both the HG001 and NA19785 samples harbor hard-to-decipher *CYP2D6-CYP2D7* fusion genes, for which Aldy only succeeded in calling a part of the *-allele. Aldy relies on literature databases to detect fusion breakpoints based on SNV definitions, which might not be discriminative enough to characterize these gene hybrids. Pangenome graph assembly-based approaches might be an attractive alternative compared to reference-based methods to elucidate the complex structure of this gene (Liao et al., 2023).

In addition, the *UGT1A1* gene poses a significant challenge for accurate diplotyping. The UGT Nomenclature committee lists 113 annotated haplotypes^4^. The Get-RM studies do not provide a consensus call for HG01190 and NA19785, despite testing on four and three different platforms, respectively. For HG001, the *1/*28 reference call is provided, while the Affymetrix platform additionally reports *60 and *93, and the Agena platform called *1/*60. The HG001 UGT1A1*28 allele was missed in our R9 flow cell data, but was detected in the R10 data, which might, as discussed before, be attributed to the improved homopolymer detection. Our Aldy results additionally indicated the presence of the *112 allele (C>A transversion) in all samples, however, A is now the reference allele, explaining the Aldy call. Therefore, this call was corrected to *1 (Supplementary Figure S8). For HG01190, the phased reads reveal the presence of the *37 allele (9 TA repeats instead of 7) missed by Aldy, probably due to limited coverage. Additionally, the *60 and *80 alleles were detected by Aldy for all samples tested, and were manually verified to be present on the same allele. In conclusion, renewed definitions of the *UGT1A1* *-alleles and further validation studies in larger cohorts are required to ascertain which alleles are truly present. Our results indicate that ONT long-reads sequencing might offer additional advantages in classifying the UGT1A locus.

For HG002 and HG005, no reference *-allele calls are available from the GeT-RM studies. We provide the major *-allele calls made by Aldy for these samples in Supplementary Table S10 for future reference.

### 4.4 Future perspectives

Alternative enrichment algorithms might be explored to further increase the throughput for our PGx panel with AS. Currently, the MinKNOW algorithm uses read mapping of the first part of the read, followed by matching this information to the provided target .bed file. Readfish is an open-source alternative to the MinKNOW algorithm and provides greater flexibility (Payne et al., 2021). The most notable improvement is the time it takes to reject a DNA strand from the nanopore. Our experiments’ average sequenced read length before a rejection decision was made was 830–880 bp, corresponding to just over 2 s of sequencing. Readfish reported shorter read N50s for the rejected reads of about 500 bp but is up to the time of writing less efficient for PromethION than for MinION/GridION due to the slower unblock time. Readbouncer, which uses k-mer counting to classify reads, claims to make faster rejection decisions than MinKNOW and Readfish, but is unsuitable for large reference databases such as whole human genomes (Ulrich et al., 2022). Recently, the BOSS-RUNS AS framework was proposed as an alternative to the AS implemented in MinKNOW. BOSS-RUNS enables to dynamically update the regions of interest based on the information already sequenced. The continuously updated target information is then passed to ReadFish, to refocus sequencing resources to, e.g., lower variant coverage sites. However, the algorithm’s computational complexity makes it unsuitable for human genomes (Weilguny et al., 2023).

Furthermore, equimolar pooling of each HMW DNA sample on a single PromethION flow cell can be difficult when the different samples’ input DNA have different size distributions (Supplementary Figure S1,S2). Shearing the DNA to a consistent input length might address this issue. However, shorter DNA fragments are disadvantageous for the AS enrichment efficiency and might result in shorter haplotype phasing blocks, which would be disadvantageous to accurately phase PGx variants. Therefore, novel approaches are emerging to tackle this pooling challenge *in silico*. Analogous to how AS enhances target genes, it can be adapted to enrich specific barcodes. While MinKNOW does not currently support the simultaneous use of AS for target genes and barcodes, barcode-aware AS with ReadFish makes this possible (Alexander et al., 2022). However, a dedicated framework to automatically balance the barcodes and achieve uniform coverage across different samples and targeted regions would be beneficial. SwordFish has been described to achieve this for SARS-CoV-2 amplicon testing and enables barcode- and amplicon balancing simultaneously. Yet, it was not demonstrated to be compatible with large-scale genomes (Munro et al., 2023).

Finally, the field of PGx itself is subject of ever-progressing knowledge into the biological consequences of genetic variation. Alternative approaches for correlating diplotypes and phenotypes are already being explored. Rather than using a categorical *-allele classification system, continuous scales have been proposed to further optimize therapeutic dosing regimens. Machine learning models can be trained to take the complete gene sequence into account to predict the resulting phenotype. These models allow to predict the effect of variants not included in a particular *-allele. The superiority of these approaches has already been shown for *CYP2D6* and validated in tamoxifen- and venlafaxine cohorts (McInnes et al., 2020; van der Lee et al., 2021). Our phased long-read PCR-free sequencing data could provide even better input for these models. However, as limitation of the current research we acknowledge that extended validation of our results in larger cohorts should be performed before clinical adoption is possible.

## 5 Conclusion

We have successfully showcased that by leveraging AS on PromethION, we can obtain comprehensive PGx data to characterize a broad panel of 1,036 PGx genes. This encompasses not just SNVs/INDELs but also structural variants, phasing, and *-allele calling. Our proposed long-read PGx diplotyping approach is not only comprehensive but also adaptable to future medical insights, making it a resilient strategy. We demonstrate that up to three DNA samples can be efficiently multiplexed on a single PromethION flow cell, improving cost-effectiveness, with recall and precision rates for targeted variants being 99.35% and 99.84%, respectively. Assigning *-alleles to the genes in our panel is currently only limited by the bioinformatical tools available to call these correctly. Future optimizations to the AS algorithms could permit multiplexing even more samples and boosting accuracy by increasing enrichment efficiency and balancing coverage across multiplexed samples. Finally, we conclude that PGx based on targeted LRS is a valuable tool to advance the implementation of personalized medicine.

## Data Availability

The datasets presented in this study can be found in online repositories. The names of the repository/repositories and accession number(s) can be found below: https://www.ncbi.nlm.nih.gov/bioproject/PRJNA1003794.

## Footnotes

1 FDA United States of America - Table of Pharmacogenomic Biomarkers in Drug Labeling. https://www.fda.gov/drugs/science-and-research-drugs/table-pharmacogenomic-biomarkers-drug-labeling2023.

2 Erasmus MC. https://www.erasmusmc.nl/nl-nl/patientenzorg/laboratoriumspecialismen/farmacogenetica#bf37da51-0bb9-441f-99de-c58eea568603.

3 Twist Bioscience. https://www.twistbioscience.com/products/ngs/Long-Read-Sequencing-Panels.

4 UGT Alleles Nomenclature Home Page. UGT Nomenclature Commitee. June 2005. http://www.ugtalleles.ulaval.ca.
