# Supplementary material for: Targeted haplotyping in pharmacogenomics using Oxford Nanopore Technologies’ adaptive sampling
Source: Front Pharmacol. 2023 Nov 13;14:1286764. doi: 10.3389/fphar.2023.1286764 (PMC10679755; doi:10.3389/fphar.2023.1286764)
Supplement: Supplementary file 1 [file DataSheet1.ZIP › SupplementaryMaterials/SupplementaryMaterials_Figures_S1-S8.pdf]

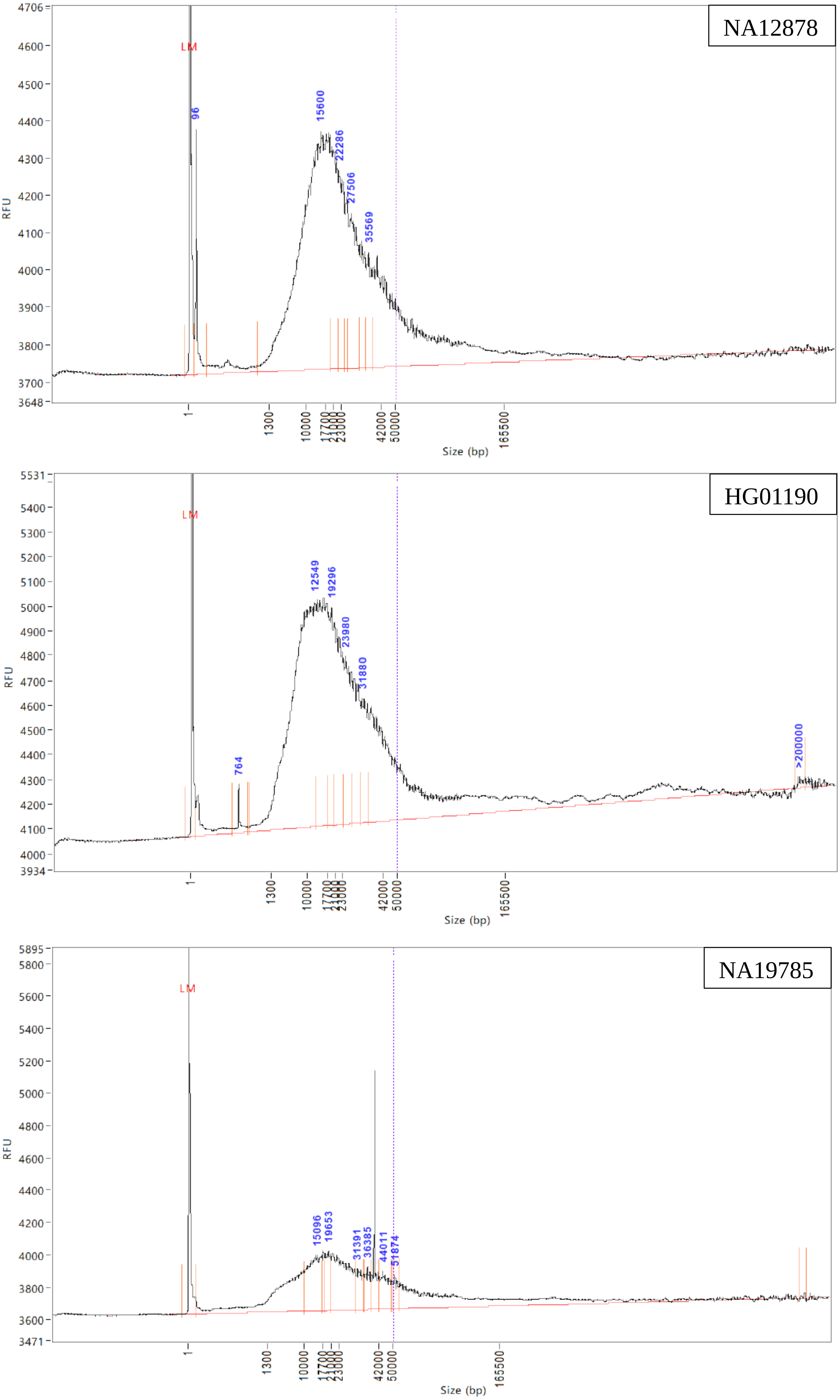

Supplementary Figure S1 Femto Pulse profiles of the NA12878 (HG001) (upper), HG01190 (middle), and NA19785 (bottom) DNA reference samples used. The x-axis displays the DNA fragment size distribution (non-linear scale). The y-axis shows the fluorescent signal relative to the amount of DNA.

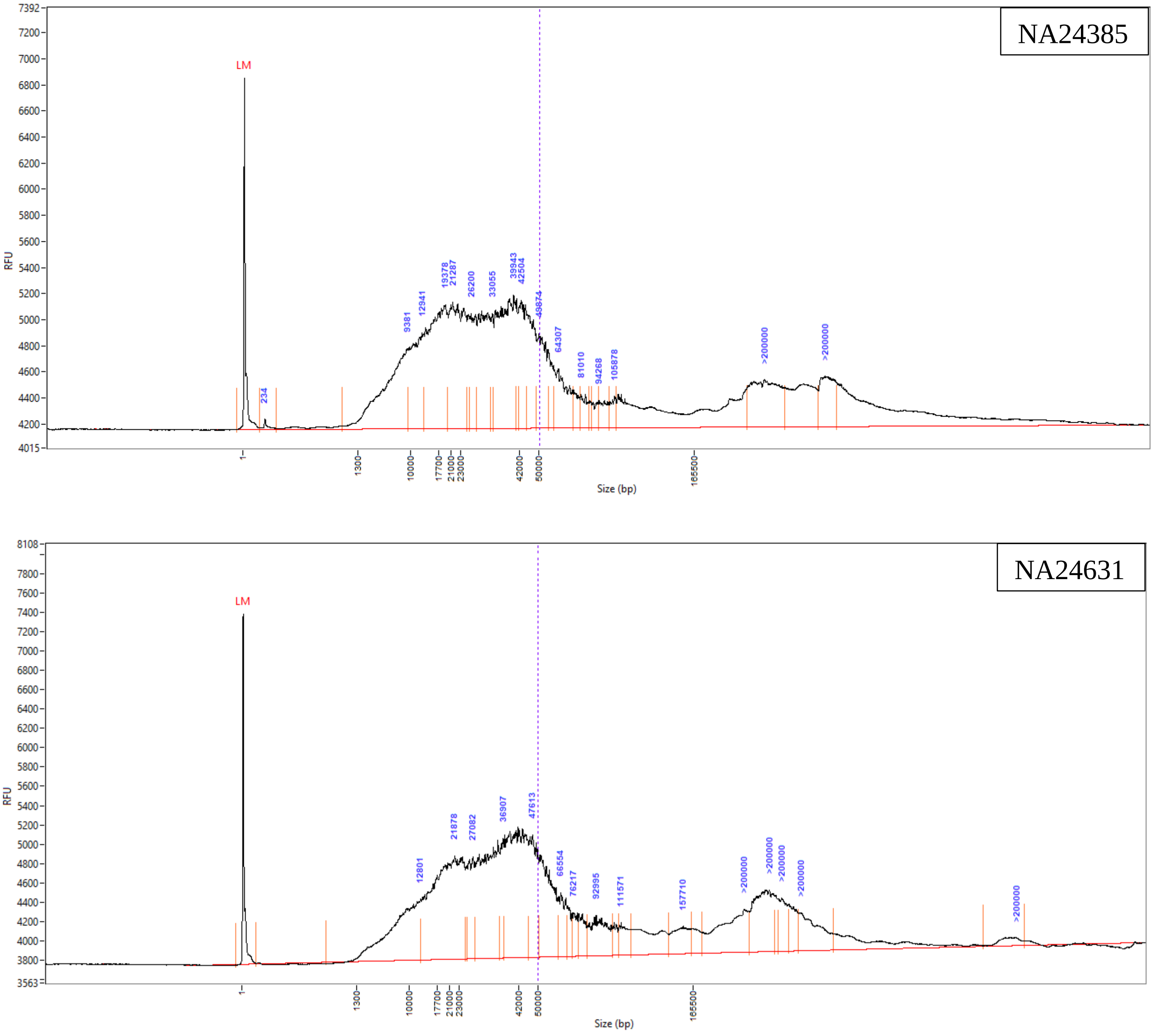

Supplementary Figure S2 Femto Pulse profiles of the NA24385 (HG002) (upper) and NA24631 (HG005) (bottom) DNA reference samples used. The x-axis displays the DNA fragment size distribution (non-linear scale). The y-axis shows the fluorescent signal relative to the amount of DNA.

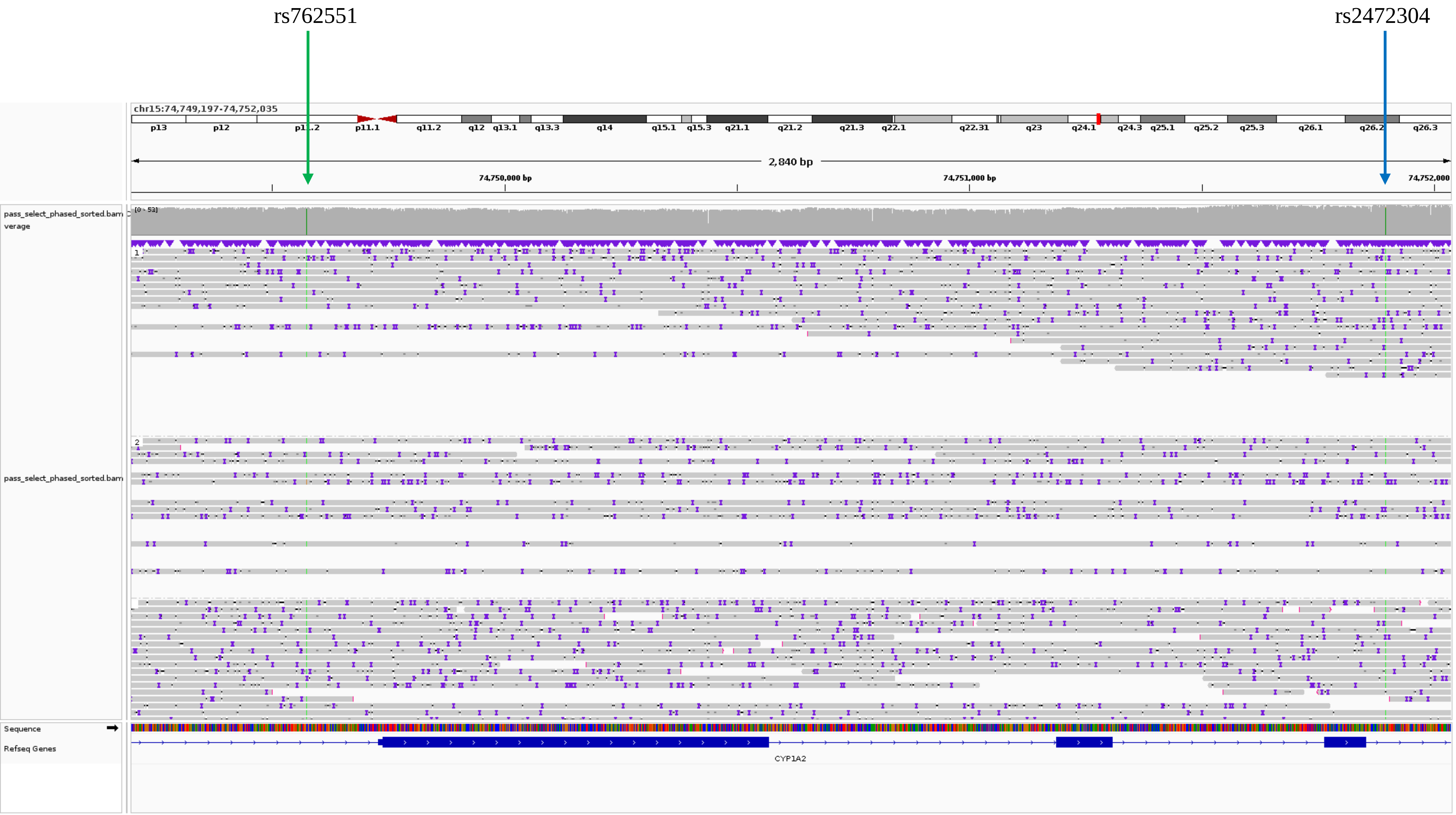

Supplementary Figure S3 IGV screenshot from the phased .bam file for the HG001 R9.4.1. data for the *CYP1A2* gene. The green arrow shows the rs762551 variant (C>A) shared by both alleles. This variant is used to distinguish the CYP1A2*1F allele according to PharmVar. The blue arrows highlights the rs2472304 variant (G>A) shared by both alleles. Together with the former variant, they make up the CYP1A2*1M allele.

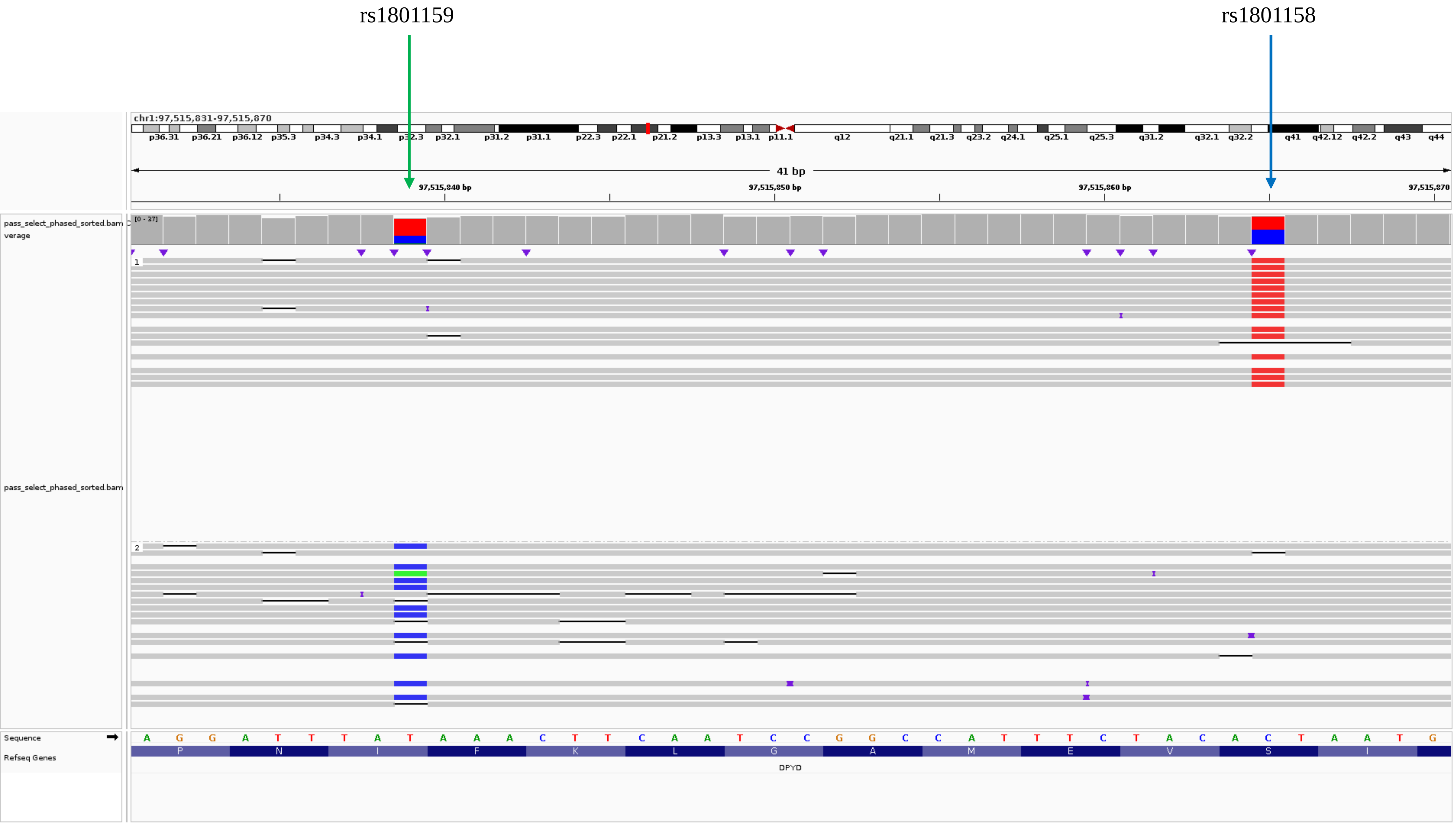

Supplementary Figure S4 IGV screenshot from the phased .bam file for the HG001 R9.4.1. data for the *DPYD* gene. The green arrow shows the rs1801159 variant (T>C) present in the bottom allele. This variant is used to distinguish the DPYD*5 allele according to PharmVar. The blue arrows highlights the rs1801158 variant (C>T) present in the top allele. This variant is used to distinguish the DPYD*4 allele according to PharmVar.

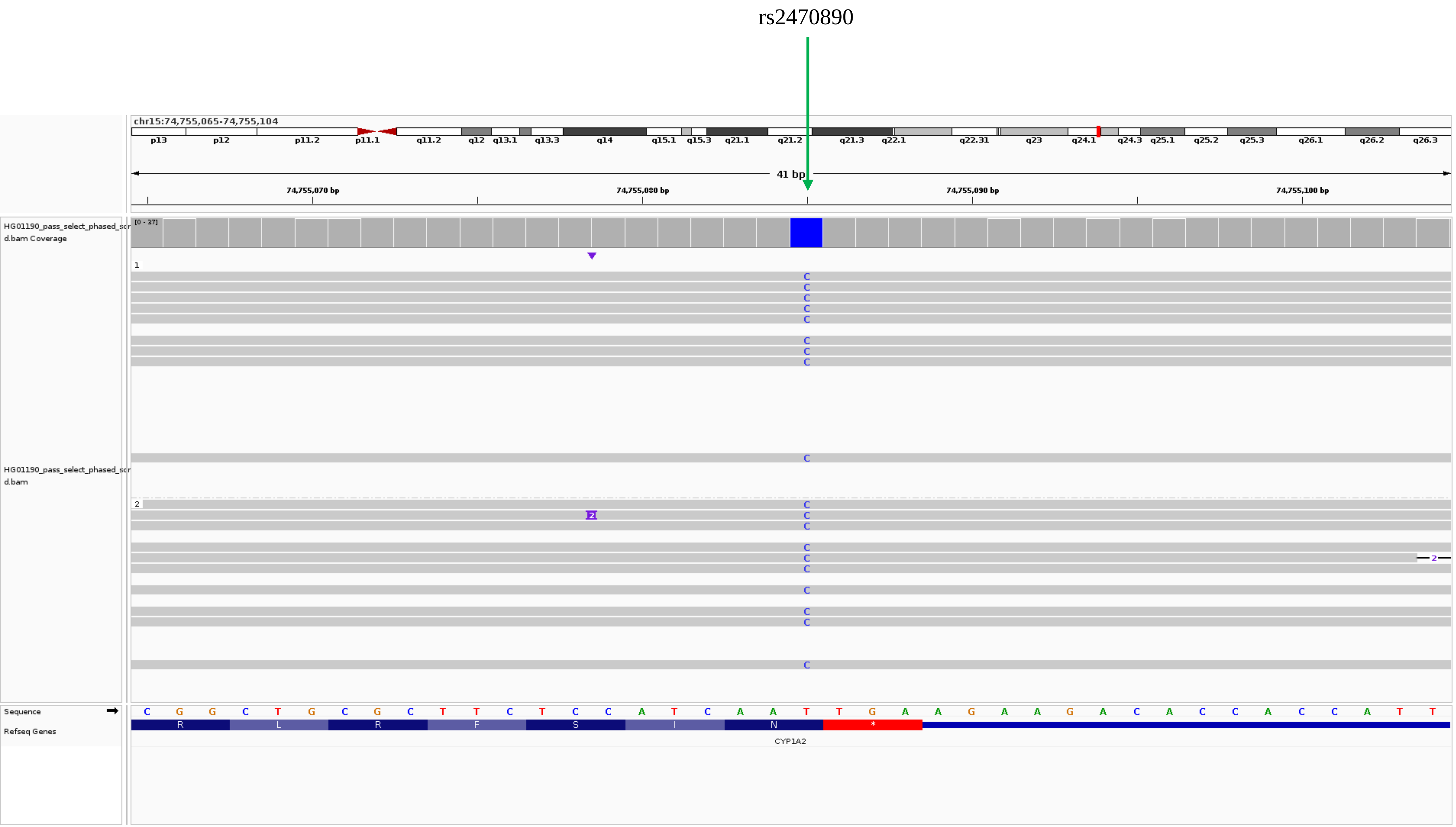

Supplementary Figure S5 IGV screenshot from the phased .bam file for the HG01190 R10.4.1. data for the *CYP1A2* gene. The green arrow shows the rs2470890 variant (T>C) shared by both alleles. This variant is used to distinguish the CYP1A2*1B allele according to PharmVar.

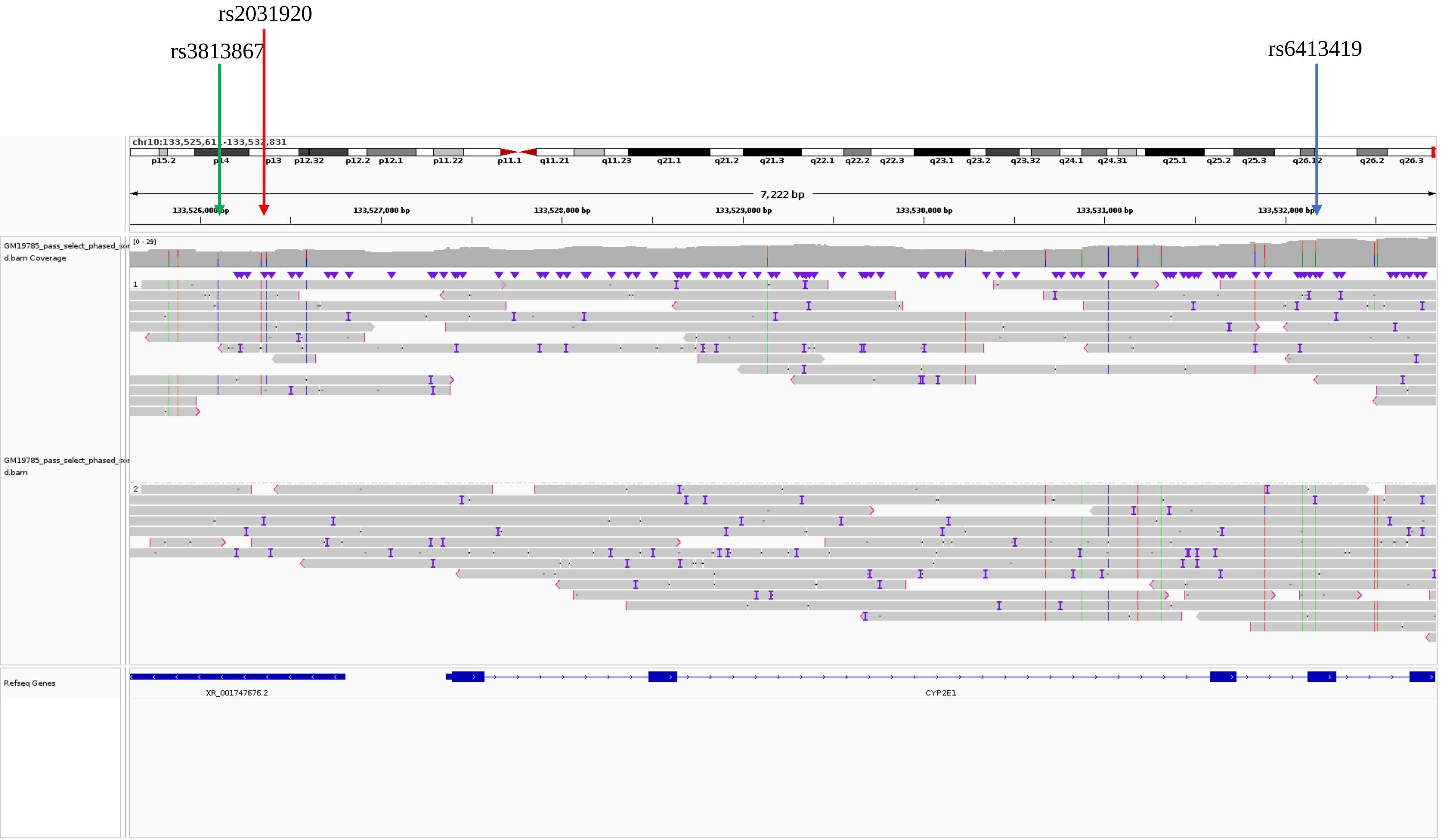

Supplementary Figure S6 IGV screenshot from the phased .bam file for the NA19785 R10.4.1. data for the *CYP2E1* gene. The blue arrow shows the rs6413419 variant (G>A) present in the bottom allele. This variant is used to distinguish the CYP2E1*4 allele according to PharmVar. The green and red arrows shows the rs3813867 variant (G>C) and the rs2031920 variant (C>T) present in the top allele, respectively. These variants are used to distinguish the CYP2E1*5 allele according to PharmVar.

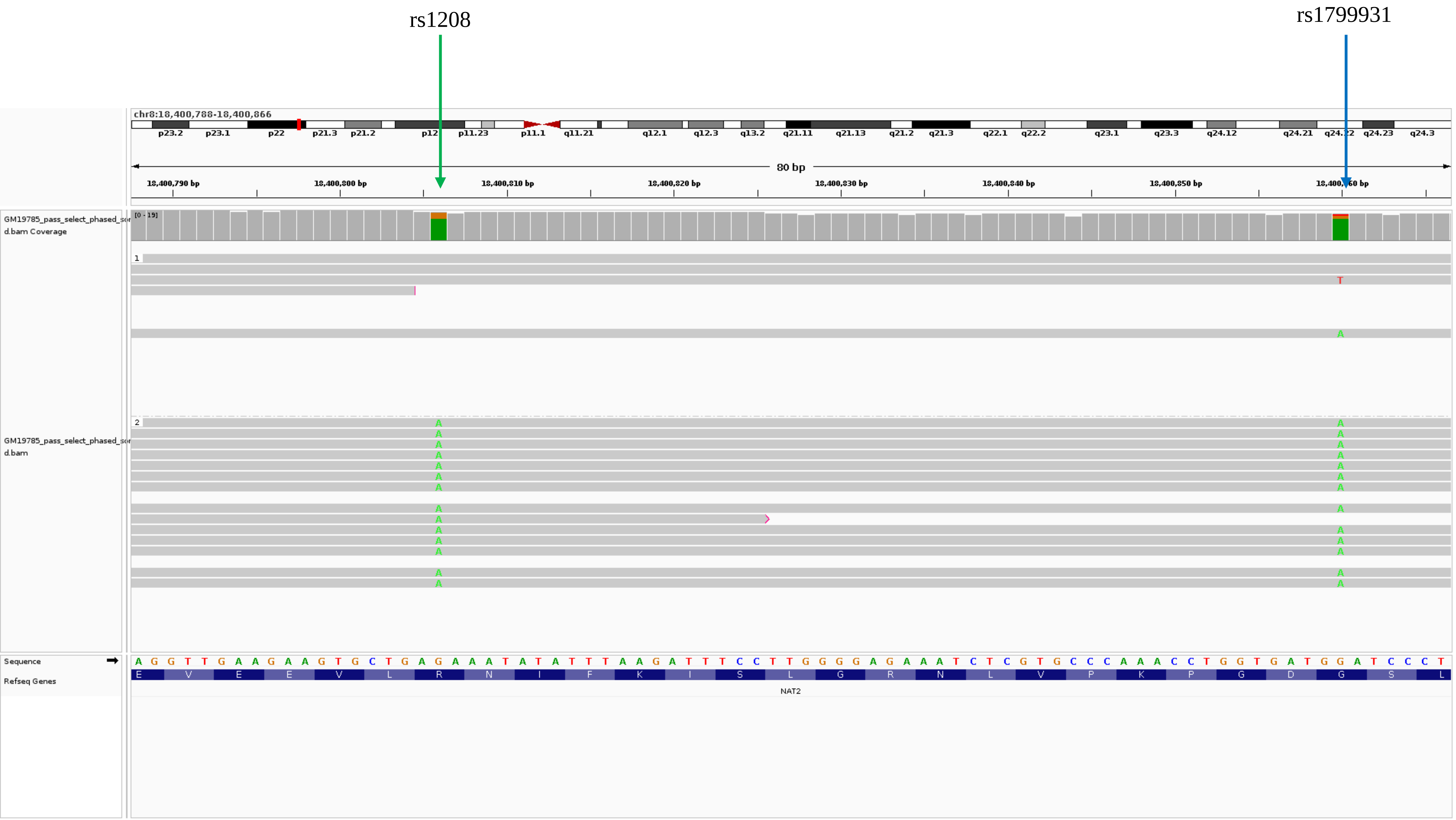

Supplementary Figure S7 IGV screenshot from the phased .bam file for the NA19785 R10.4.1. data for the *NAT2* gene. The green arrow shows the rs1208 variant. The Arylamine N-acetyltransferase Gene Nomenclature Committee denotes this variant as an A > G transition. However, the current GRCh38 genome contains a guanine nucleotide at this position, resulting in the National Center for Biotechnology Information defining it as a G>A, G>C or G>T variant. As the top allele has a guanine nucleotide on this position, we assign *12, in line with the currently still used Arylamine N-acetyltransferase Gene Nomenclature Committee. The blue arrow shows the rs1799931 variant (G>A) present in the bottom allele. This variant is used to distinguish the NAT2*7 allele.

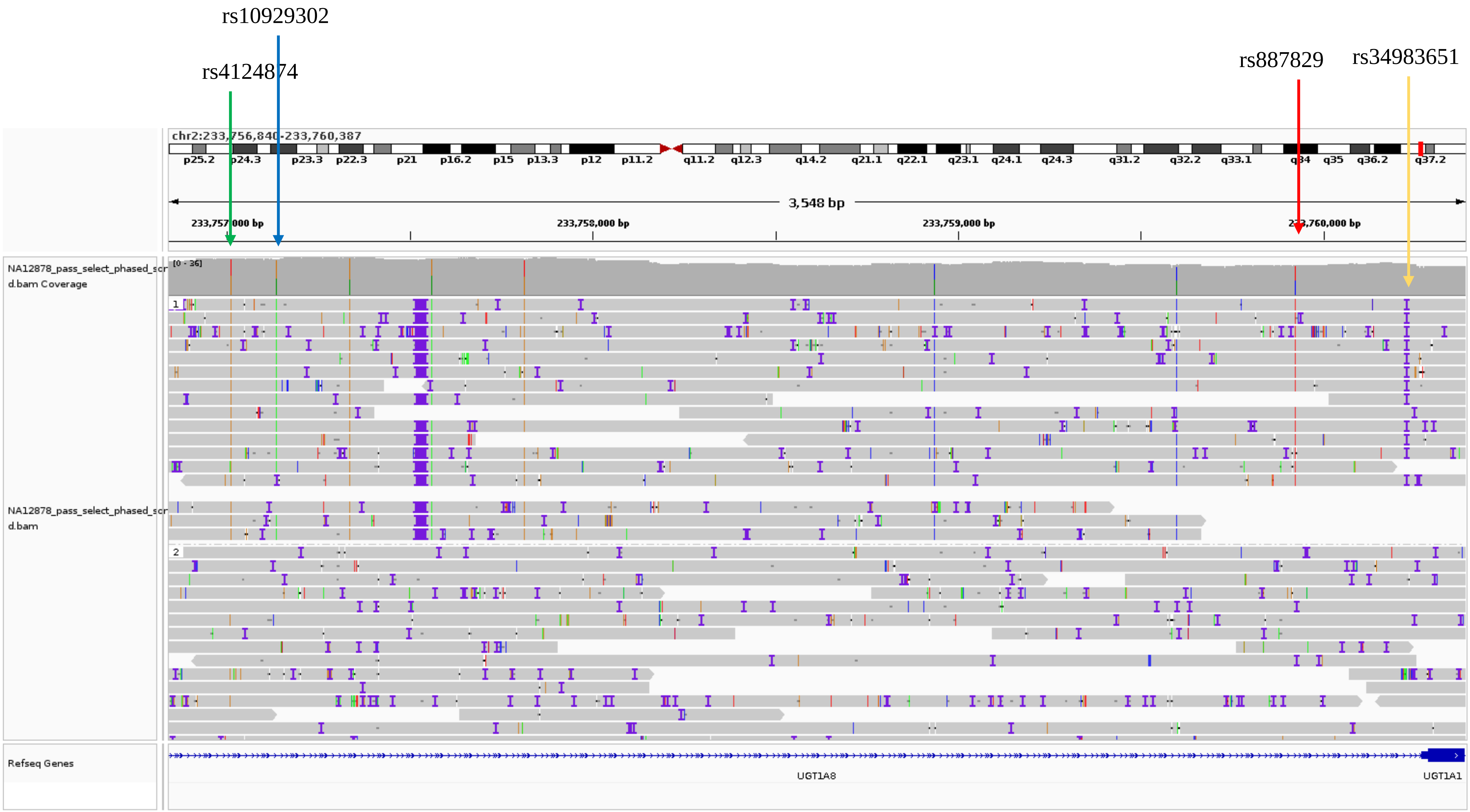

Supplementary Figure S8 IGV screenshot from the phased .bam file for the HG001 R10.4.1. data for the *UGT1A1* gene. The green, blue, red, and yellow arrows show the rs4124874 (*60), rs10929302 (*93), rs887829 (*80), and rs34983651 (*28) variants, respectively. All variants are located on the same allele, and are used by the UGT Nomenclature committee to assign the *28+*60+*80+*93 allele. The bottom allele is assigned as the reference, i.e. *1.
